# Novel MRSA-targeting phage MetB16: Genomic features, structural insights, and therapeutic applications

**DOI:** 10.55730/1300-0152.2746

**Published:** 2025-02-14

**Authors:** Berna ERDOĞDU, Senanur DOKUZ, Görkem GÜNGÖR, Wei LIN, Yigang TONG, Tülin ÖZBEK

**Affiliations:** 1 Department of Molecular Biology and Genetics, Faculty of Arts and Science, Yildiz Technical University, İstanbul, Turkiye; 2 College of Life Science and Technology, Beijing University of Chemical Technology, China

**Keywords:** Methicillin-resistant *Staphylococcus aureus* (MRSA), prophage induction, modeling phage protein, phage bioinformatics

## Abstract

**Background/aim:**

Recent reports have indicated that multidrug-resistant strains of S. aureus, including methicillin-resistant strains, may pose a significant threat to public health and global economic stability.

**Materials and methods:**

In this study, we present the isolation and comprehensive characterization of a novel phage, derived from clinically isolated MRSA strains.

**Results:**

MetB16 exhibited an incubation period of approximately 20 min, a lysis period of around 45 min, and a burst size of 127 Plaque Forming Units (PFU)/cell. The phage demonstrated remarkable biological stability across a pH spectrum of 4.0–9.0 and maintained integrity within a temperature range of 37 and −80 °C. Scanning transmission electron microscopy and phylogenetic analyzes classified MetB16 as belonging to the Triavirus genus, representing a novel species within the Triaviruses. Whole-genome sequencing revealed a 45,295 bp-long genome size with a G + C content of 33.34%. Notably, bioinformatic analyses identified random integration sites within the MRSA genome. Functional annotation of the genome uncovered 72 open reading frames (ORFs), of which 34 encoded hypothetical proteins of unknown function, and these ORFs were associated with phage structure, packaging, host lysis, DNA metabolism, and additional functions. To elucidate the therapeutic potential of temperate phages, detailed structural analyses were conducted on key proteins, including holin, endolysin, and minor tail proteins of MetB16.

**Conclusion:**

This study provides for the first time, the preliminary studies on the biological properties of MetB16 and comprehensive data facilitating an in-depth analysis of the mechanism underlying phage-host interactions, serving as a valuable reference for the evaluation of temperate phages in phage therapy.

## Graphical Abstract

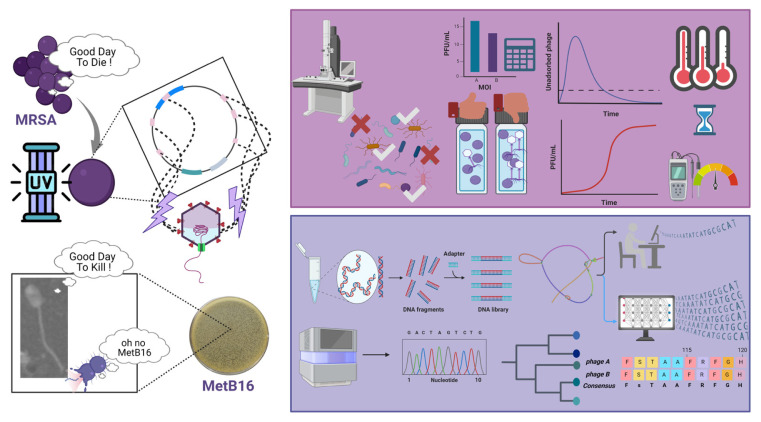

## 1. Introduction

*Staphylococcus aureus*, a Gram-positive pathogen bacteria from *Staphylococcaceae* family, is a common commensal organism, asymptomatically colonizing various parts of the human body, particularly the nasal passages of approximately 20%–30% of individuals (Wertheim et al., 2005; Gherardi, 2023). While typically benign, *S. aureus* colonization increases the risk of subsequent infections, which can range from minor skin and soft tissue infections to severe conditions such as osteomyelitis, bacteremia, pneumonia, and endocarditis (Diekema et al., 2001; Clarridge Iii et al., 2013). These infections may manifest acutely or persist as chronic or recurrent conditions (Howden et al., 2023). The widespread use of antibiotics initially proved effective in controlling bacterial infections; however, selective pressure has driven the evolution of resistance mechanisms in bacteria like *S. aureus* (Ma et al., 2020; Magalhães et al., 2021). A key factor in this evolution has been the horizontal transfer of (Staphylococcal Cassette Chromosome mec) SCCmec elements, which has endowed *S. aureus* with resistance to methicillin and other β-lactam antibiotics, leading to the emergence of methicillin-resistant *Staphylococcus aureus* (MRSA) (Peacock and Paterson, 2015; Hassoun et al., 2017). Since its rapid spread in the late 1980s, MRSA has become a significant clinical concern, challenging conventional anti-infective therapies (Lakhundi and Zhang, 2018; Turner et al., 2019). This rising threat underscores the urgent need for novel therapeutic strategies to combat MRSA.

Phages are supramolecular and highly adaptable entities capable of infecting their hosts by encapsulating their nucleic acids within protein capsids (Hatfull and Hendrix, 2011; Ge et al., 2021). However, the application of existing phages is often constrained by challenges related to isolation, culturing, infection mechanisms, and the development of phage resistance (Clokie et al., 2011; Skanata and Kussell, 2021). Moreover, a comprehensive evaluation of phages with distinct life cycles is essential to fully exploring their therapeutic potential. Lysogenic phages exhibit significant diversity and widespread distribution, often found integrated as prophages within the genomes of numerous sequenced bacteria (Nadeem and Wahl, 2017; Gonçalves et al., 2021; Greenrod et al., 2022). Traditionally regarded as having limited bactericidal capabilities, temperate phages face obstacles such as superinfection immunity resulting from gene integration, which diminishes the susceptibility of host bacteria to subsequent phage attacks (Dedrick et al., 2017). However, horizontal gene transfer (HGT) via lysogenic phages facilitates the acquisition of mobile genetic elements, including toxin genes, virulence island gene clusters, and antibiotic resistance genes, further enhancing bacterial virulence and complicating treatment (Waldor and Mekalanos, 1996; Shaikh and Tarr, 2003; Chen and Novick, 2009; Haaber et al., 2016). Additionally, recent advances in high-throughput sequencing and synthetic biology, improved phage genome knowledge and engineering strategies have changed the outlook for temperate phages with a lysogenic life cycle (Monteiro et al., 2019). Some studies have demonstrated that temperate phages can be induced to acquire lytic properties through targeted mutations and that the natural loss of lysogenic genes may also occur (Mahler et al., 2023; Shan et al., 2023). Increasing the knowledge related to temperature phages is important for providing critical insights into the intricate coevolution of phages, bacteria, and their human hosts.

In this study, we report the isolation and characterization of a novel phage, vB_Sau_MetB16 (MetB16), derived from clinical MRSA strains. For the first time, we present preliminary insights into the biological properties of MetB16, including its lytic activity, growth dynamics, and stability. Additionally, we performed comprehensive analyses of its whole genome and proteome, providing a detailed understanding of its molecular architecture.

## 2. Materials and methods

### 2.1. Bacterial strain and culture condition

Clinically isolated methicillin-resistant *Staphylococcus aureus* (MRSA) strain were stored in Luria-Bertani (LB) broth medium containing 25% glycerol at −80 °C. MRSA were inoculated on LB agar medium and cultured at 37 °C overnight. Colonies grown on LB agar were stored at 4 °C to be used in studies (Li et al., 2020). *S. aureus* RN4220; *S. aureus* (ATCC 25923); Penicillin (P) and Amoxicillin (AM) resistant *S. aureus*; P, AM and Trimethoprim/Sulfamethoxazole (SXT) resistant *S. aureus*; clinically isolated MRSA; MRSA (ATCC 67101); Oxacillin susceptible *S. aureus*; *S. aureus* (ATCC 6538); Imipenem (IMI) resistant *Acinetobacter baumannii*; *Bacillus subtilis* (ATCC 6633); *Pseudomonas aeruginosa* (ATCC 27853); *Escherichia coli* (ATCC 25922); *Enterococcus faecalis* (ATCC 29212); clinically isolated *Streptococcus mutans*; P and AM resistant *S. simulans;* Enterohemorrhagi*c E. coli;* FOX, P and AM resistant *S. epidermidis, S. chromogenes* were cultured overnight at 180 rpm in LB broth at 37 °C.

### 2.2. Prophage isolation

MRSA cultures were grown overnight at 37 °C in LB broth with agitation 180 rpm. For the induction experiment, 100 μL of the overnight culture was inoculated into 10 mL of fresh LB broth and incubated under the same conditions until the cultures reached an optic density (OD)600 of 0.5–0.6. At this point, the cultures were exposed to UV light for varying durations (30, 45, 60, 90, 120, and 150 s) to induce prophage excision from the MRSA genome. Postexposure, the cultures were incubated for an additional 4 h under the same temperature and shaking conditions. The cultures were then centrifuged at 5000 × g for 3 min, and the resulting supernatant was filtered through a 0.22 μm filter to obtain the phage filtrate (Abraha et al., 2022; Wang et al., 2023). To detect the presence of phages in the induced filtrates, a spot assay was conducted, followed by the double agar overlay method for quantification. In the spot assay, *S. aureus* RN4220 was prepared at a concentration of 10^8^ Colony Forming Unit (CFU)/mL, and 100 μL of the culture was swabbed onto Brain Heart Infusion (BHI) agar plates. After preincubation at 37 °C for 1 h, 10 μL of phage filtrates were spotted onto the plates. Following overnight incubation at 37 °C, lysis zones were assessed, and the filtrate with the clearest profile was selected for further analysis (MetB16). The phage titer, expressed in plaque-forming units (PFU), was calculated using the double agar overlay method according to the following formula.


★PFU/mL=Number of plaques/(Dilution factor×Volume of filtrate added in mL)

For the assay, 100 μL of diluted phage filtrate was mixed with 100 μL of *S. aureus* RN4220 suspension (10^8^ CFU/mL) in BHI broth. After a 30 min incubation at room temperature, 2 mL of BHI top agar was added, gently vortexed, and poured onto a 10 mL BHI bottom agar plate. Plates were dried at room temperature for 10 min before being inverted and incubated overnight at 37 °C. To purify a single phage, a selected plaque was picked using a sterile wooden stick, and the double agar overlay technique was repeated five times. For enrichment, the lysis zones were scraped from the agar and transferred into a broth medium containing host bacteria cultured for 4 h. After a 10 min incubation at room temperature, fresh broth was added, and the mixture was further incubated at 37 °C for 18 h. The culture was then centrifuged at 5000 × g for 10 min, and the supernatant was filtered (Selcuk et al., 2024b).

### 2.3. Structural morphology

The phage filtrate, at a concentration of 10^9^ PFU/mL, was assessed using Scanning Transmission Electron Microscopy (STEM) with a Thermo Scientific Quattro S, offering a resolution of 0.8 nm. For imaging, samples were prepared by negative staining with a 1% uranyl acetate solution. The stained samples were then applied to carbon-coated grids for observation. STEM analysis was conducted at an accelerating voltage of 80 kilovolts (kV), with magnification set to 180,000X to facilitate a detailed examination of the phage structure (Selcuk et al., 2024b,a).

### 2.4. Host range determination

The host range of the induced prophage were determined by spot test and double agar overlay method described earlier. Twenty-one different bacterial strains were tested for their susceptibility to the phage by incubating them with the phage and observing plaque formation (Selcuk et al., 2024b). The efficiency of plating (EOP) of the phage was calculated by dividing the average phage titer on the nonhost bacteria by the phage titer on the host bacterium, which serves as the reference with an EOP value of 1 (Ngiam et al., 2022).

### 2.5. Multiplicity of infection assay (MOI)

To determine the optimal MOI, exponential phase host bacteria (10^6^ CFU/mL) were mixed with diluted phages at MOIs of 0.001, 0.01, 0.1, 1, 10, and 100. The mixtures were incubated at 37 °C for 6 h with shaking. Postincubation, samples were centrifuged at 10,000 × g for 6 min to remove precipitates. The supernatants were filtered, and the phage titer was assessed using the double agar overlay method. The MOI yielding the highest phage titer was identified as optimal (Selcuk et al., 2024b).

### 2.6. Adsorption curve and one-step growth analysis

For adsorption rate measurement, 2.5 mL of phage (10^8^ PFU/mL) and 2.5 mL of host bacteria (10^8^ CFU/mL) were mixed (MOI=1) and incubated at 37 °C with shaking. At intervals (0, 2, 5, 10, 15, 20 min), samples were taken and centrifuged at 12,000 × g for 2 min. In the one-step growth analysis, the phage (10^8^ PFU/mL) and host bacteria (10^8^ CFU/mL) were mixed (MOI = 1) and incubated at 37 °C for 10 min to allow adsorption. Following centrifugation at 12,000 × g for 2 min to remove unadsorbed phages, the pellet was resuspended in 10 mL of BHI broth. At determined time points (0, 5, 10, 15, 20, 25, 30, 35, 40, 45, 50, 55, 60 min), samples were collected and phage titers were measured using the double agar overlay method. The burst size was calculated by dividing the average titers at postburst time points by the average initial titers (Peng et al., 2020; Tao et al., 2021; Selcuk et al., 2024b)

### 2.7. Stability testing

The stability of the phage was evaluated under various temperature conditions (−80 °C, −20 °C, 4 °C, 25 °C, 37 °C, 50 °C, 60 °C, and 70 °C) and across a wide pH range (1 to 14). For temperature stability, 10^8^ PFU/mL of the phage filtrate was incubated in BHI broth medium at the specified temperatures for 1 h. pH stability was assessed by incubating the phage filtrates in phosphate-buffered saline (PBS) solutions with pH values ranging from 1.0 to 12.0 for 1 h. The phage titers were then determined using the double agar overlay method (Selcuk et al., 2024a).

### 2.8. Genome sequencing, assembly and annotation

DNA isolation of the bacteria and phage were respectively carried out using the Genomic DNA Isolation Kit (GenedireX, Inc.) and Phage DNA Isolation Kit (Norgen Biotek Corporation, Thorold, Canada) according to the manufacturer’s instructions. Subsequently, the quantity of isolated DNA was assessed by nanodrop spectrophotometer (Thermo Scientific Invitrogen Nanodrop One Spectrophotometer) and stored at −20 °C for a short time. Phage whole-genome sequencing was performed using the Oxford Nanopore Technologies (ONT, Oxford, UK) MinION (Mk1B) instrument, and the read files were assembled using SPAdes software (v3.13.1) with default parameters, according to Selcuk et al.’s protocol.(Jain et al., 2016; Selcuk et al., 2024b). The sequencing reads were aligned to the phage genome using CLC Genomics Workbench (version 20.0.4), applying matching thresholds of length fraction >0.25 and similarity fraction >0.8. Subsequently, the single assembled contig was annotated using the Rapid Annotation using Subsystem Technology Toolkit (RASTtk) pipeline from the BV-BRC tool. (Brettin et al., 2015; Mcnair et al., 2019). The annotation scheme commenced with “annotate-proteins-phage”, followed by “annotate-proteinskmer-v2”. tRNAscan-SE (v2.0) was utilized for identifying tRNA genes (Chan and Lowe, 2019). After the initial RASTtk annotation, a subsequent round of annotation was carried out to either verify assigned functions or assign functions to proteins without annotations. Several tools were utilized for this task, including UniProt, NCBI BLASTp, InterPro Scan, and HHPred. The genomic map of the phage was generated using the Proksee web-based tool (Grant et al., 2023) which belongs to the CGView family of tools (Stothard et al., 2018).

### 2.9. Bioinformatics analysis and comparative genomics

The phage lifestyle was predicted using PhageAI platform. PhageLeads was employed to identify temperate genetic markers, antimicrobial resistance (AMR) factors, and virulence genes (Yukgehnaish et al., 2022). Suitability for therapeutic applications was assessed with the RGI Resistance Gene Identifier (RGI v5.2.1, CARD v3.2.3) and VRprofile2 (Wang et al., 2022). PhageTerm (Galaxy v1.0.11) was used to predict genome termini and packaging patterns from sequence reads (Garneau et al., 2017). Specific phage proteins were analyzed for transmembrane domains using DeepTMHMM (v1.0.24) (Hallgren et al., 2022). The genomic sequence was further examined using the Phage Depolymerase Finder (PhageDPO) (Galaxy Version 0.1.0) to identify potential depolymerase genes (Vieira et al., 2023). Genomic comparisons were performed with sequences in the GenBank-NCBI database using BLASTn, focusing on sequences with ≥90% coverage and identity. Average nucleotide identity (ANI) was calculated using JSpeciesWS (Richter et al., 2016), and proteome comparisons were conducted using the BV-BRC Proteome Comparison tool with BLASTp analysis (Davis et al., 2020).

Genome-based taxonomy was conducted according to Shibiny et al. (Zaki et al., 2023). Family-level classification was performed using the viral proteomic tree server (ViPTree), which employs proteome-based clustering (Nishimura et al., 2017). Phages with the highest tBLASTx scores from ViPTree were selected to construct a detailed rectangular proteomic tree. For more precise classification below the family level, we utilized the Virus Classification and Tree Building Online Resource (VICTOR), applying the Genome-BLAST Distance Phylogeny (GBDP) method (Meier-Kolthoff and Göker, 2017). Analysis included 100 pseudo-bootstrap replicates, with trees rooted at the midpoint and visualized using ggtree (Yu, 2020). Taxon boundaries were estimated using the OPTSIL program (Göker et al., 2009), with clustering thresholds and an F value of 0.5 (Meier-Kolthoff et al., 2014). Intergenomic similarities between the isolated phage and closest homologs were assessed using Virus Intergenomic Distance Calculator (VIRIDIC), which calculates pairwise distances/similarities according to ICTV guidelines. Similarity percentages were visualized with a heatmap (Moraru et al., 2020).

### 2.10. Modeling of therapeutic proteins

To explore the therapeutic and targeting potential of temperate phage proteins, we performed a functional analysis of the holin, endolysin, and minor tail protein of phage MetB16 using Interpro (Paysan-Lafosse et al., 2023). Subsequently, 3D structures of these proteins were modeled with AlphaFold 2 (AF2) (Jumper et al., 2021), utilizing default parameters including pdb100 for template mode, num_relax set to 1, and auto-adjusted model type and num_recycles. The quality of these models was evaluated with MolProbity (Williams et al., 2018) and the Swiss-Model Structure Assessment tool (Benkert et al., 2011).

The genomic sequences of phage MetB16 and the host bacteria have been deposited in the NCBI nucleotide database under accession numbers PP357935 and CP162608, respectively.

## 3. Results

### 3.1. Prophage isolation and characterization

**Figure 1:**
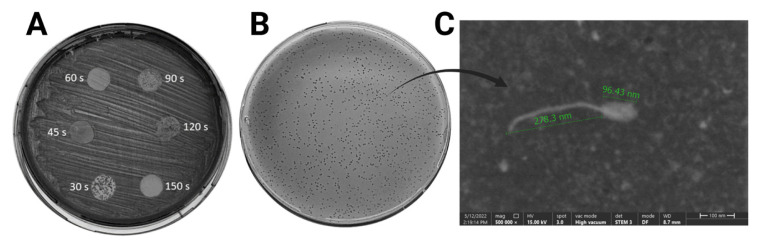
The spot test results (A) of phage filtrates obtained after specified durations of UV exposure of MRSA cultures, the plaque morphology of the selected phage filtrate (B), and STEM analysis (C) are presented.

The results of the spot test following UV exposure of MRSA cultures at various intervals are shown in Figure 1A. Prophage induction was evident across all UV exposure times, though not all lysis zones were clean. The phage filtrate from the 60-s UV exposure exhibited a notably clear and smooth lysis zone, and thus was chosen for further analysis. After selecting single plaques via the double agar overlay method, phage enrichment was performed to boost the titer. The STEM image of MetB16, presenting as clear, small plaques approximately 2–5 mm in diameter (Figure 1B), is depicted in Figure 1C. Morphological analysis confirmed that MetB16 belongs to the *Caudoviricetes* class, characterized by its icosahedral head and long tail. The phage particle features an isometric head with a diameter of 96.43 ± 5.4 nm and an overall length of 374.73 ± 10.3 nm.

**Figure 2:**
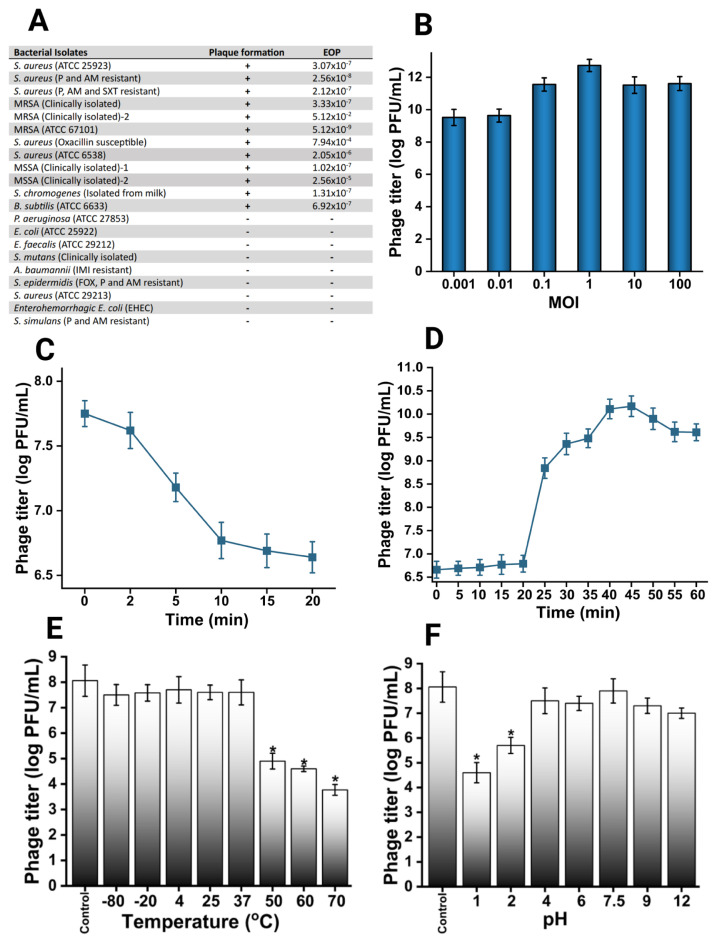
The results of the host range (A), optimal MOI (B), adsorption curve (C),one-step growth graph (D), stability under certain temperature (E) and pH values (F) are shown. Statistically significant differences determined by the Friedman test were marked with an asterisk (*): p < 0.05.

The host range specificity of MetB16 was evaluated through a spot test, with positive isolates subsequently tested for plaque formation using the double agar overlay method (Figure 2A). MetB16 demonstrated lytic activity against all tested strains of *S. aureus, S. chromogenes*, and *B. subtilis.* The optimal multiplicity of infection (MOI) for MetB16 was determined to be 1, based on the highest phage titer achieved (Figure 2B). The adsorption curve analysis showed that the phage quickly bound to the bacterial surface, reaching peak adsorption by 20 min (Figure 2C). One-step growth analysis revealed a latent period of approximately 20 min, a burst phase extending up to 45 min, and an average burst size of 127 PFU/mL (Figure 2D). MetB16 retained stability and lytic activity effectively across a temperature range from −80 to 37 °C (Figure 2E), but significant declines in viability and activity were noted at temperatures above 50 °C. Additionally, MetB16 maintained a relatively high survival rate within a pH range of 2.0 to 9.0, with the highest phage titer observed between pH 4.0 and 9.0, approximately 10^7–8^ PFU/mL.(Figure 2F).

### 3.2. Sequencing and annotation of the phage genomes

After DNA extraction, library preparation, and ONT sequencing, a total of 126,049 single-end sequence reads were generated. The data yielded an N50 of 1383 bp, with the longest read at 34,127 bp and the shortest at 70 bp, amounting to a total of 107.3 Mbp. The reads were assembled into a single contig of 45,295 bp, with an average read coverage of 104 and a GC content of 33.34%. Assembly was performed using SPAdes. To explore phage integration regions in the bacterial genome, raw reads were mapped to the MetB16 genome. Figure S1 illustrates that many reads partially align with the phage sequence (dark lines), while others do not (light lines). Further BLASTn analysis (Table S1A) revealed that the unmatched sequences originated from the *S. aureus* genome, indicating a random rather than fixed integration pattern for MetB16.

PhageTerm analysis indicated that MetB16 features a terminally redundant genome with partial circular permutations, consistent with the headful packaging mode. This mode involves random cleavage of linear concatemeric DNA during assembly to package the genome into the capsid, often resulting in a genome length exceeding the actual size by over 100%.

**Figure 3:**
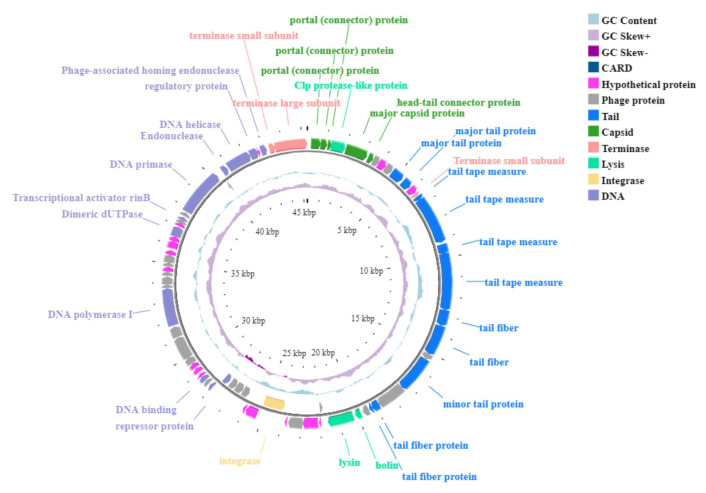
Genomic circular map of phage MetB16. Various colors are used to represent the coding sequences (CDS) based on their predicted functions: phage proteins (grey), terminase (salmon pink), capsid proteins (green), lytic enzymes (light green), tail proteins (blue), DNA metabolism, replication, repair, and binding-related proteins (light purple), hypothetical proteins (pink) and Integrase gene (orange). The GC content skew is depicted in the middle circle with a light blue color, while the inner circle illustrates the GC skew using lilac and dark purple colors for above and below averages, respectively.

Genome annotation was performed using BV-BRC’s PHANOTATE tool, followed by manual curation. Seventy-two open reading frames (ORFs) were predicted, with 34 assigned known functions (Table S1B, Figure 3). Protein sequences were analyzed using various tools, with genes of known function highlighted in blue and hypothetical proteins marked in gray (Table S1B). Among the predicted ORFs, 2 began with the TTG codon, 9 with GTG, and the remainder with ATG. Except for 7 genes related to integrase, repressor, and phage functions, all other ORFs with predicted functions were oriented on a single strand. Among the ORFs with unknown functions, 24 matched other phage proteins, while 13 have not yet been confirmed. The GC content was calculated at 33% using the geecee tool. Annotated genes with known functions were categorized into five groups: DNA replication, repair, transcription, and binding (12 genes); virion structure (15 genes, including 4 capsid-associated and 11 tail-associated genes); genome packaging and assembly (5 genes); and host cell wall lysis (2 genes) (Table S1B).

Structural protein-coding genes are classified into capsid and tail categories. Capsid genes, responsible for portal and major proteins, are located between nucleotide positions 195 and 3,517, with intervening genes encoding endopeptidase. Genes responsible for tail structure or function, such as tape measure, major-minor tail, and tail fiber proteins, are situated from nucleotide positions 4690 to 19344. The tail spike protein (ORF 18) exhibits depolymerase activity, as predicted by the PhageDPO tool with 76% accuracy (Table S2). Genome packaging and assembly genes include small and large terminase proteins, along with a phage-associated homing endonuclease. The genes involved in lysis, including holins, and endolysin, span nucleotide positions 19,825 to 21,591 without intergenic regions. DeepTMHMM analysis confirms the presence of class II holins in the putative holin protein (ORF 26), with a transmembrane topology probability of 100% (Figure S2). PhageAI classifies MetB16 as temperate, suggesting a lysogenic lifecycle. Comprehensive genomic screening with tools like ResFinder (v4.1), VRprofile2, and RGI v5.2.1 revealed no virulence or antimicrobial resistance genes.

### 3.3. Comparative genomic and proteomic analyses

**Figure 4:**
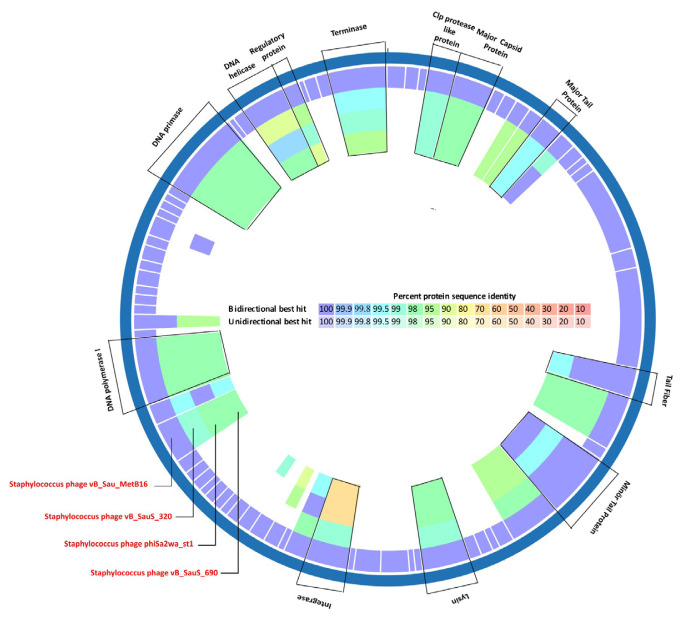
Circular comparative map of protein-coding genes between phage MetB16 and closely related phages (Staphylococcus phage phiSa2wa_st1, Staphylococcus phage vB_SauS_320, and Staphylococcus phage vB_SauS_690).

To explore the phylogenetic neighborhood of phage MetB16, we conducted a comprehensive screening of publicly available NCBI nucleotide data (GenBank) using BLASTn. This analysis identified 30 phages with substantial genomic similarity, showing sequence identities greater than 96% and covering 85% to 93% of their genomes (Table S3). These phages were classified within the *Siphoviridae* family and further categorized into the genus *Triavirus*. Among the 30 phages, comparison with MetB16 revealed ANI values exceeding 95% in global alignment. The three phages with the highest ANI values were *Staphylococcus* phage phiSa2wa_st1, Staphylococcus phage vB_SauS_320, and *Staphylococcus* phage vB_SauS_690 (with NCBI accession numbers NC_055045.1, OM439665.1, and OM439667.1, respectively) (Table S4). Proteomic comparison using BV-BRC tools indicated high levels of protein-coding gene orthology, with amino acid sequence identities surpassing 95% (Table S5). Orthologous proteins include Clp protease-like protein (ORF 4), major capsid protein (ORF 5), major tail protein (ORF 10), tail fiber (ORF 18), minor tail protein (ORF 21), lysin (ORF 27), DNA polymerase I (ORF 49), DNA primase (ORF 64), DNA helicase (ORF 67), regulatory protein (ORF 68), and terminase (ORF 72) (Figure 4). Notably, the integrase (ORF 33) of MetB16 showed closer similarity to that of OM439665.1 compared to NC_055045.1 and OM439667.1.

### 3.4. Phylogenetic analysis

**Figure 5:**
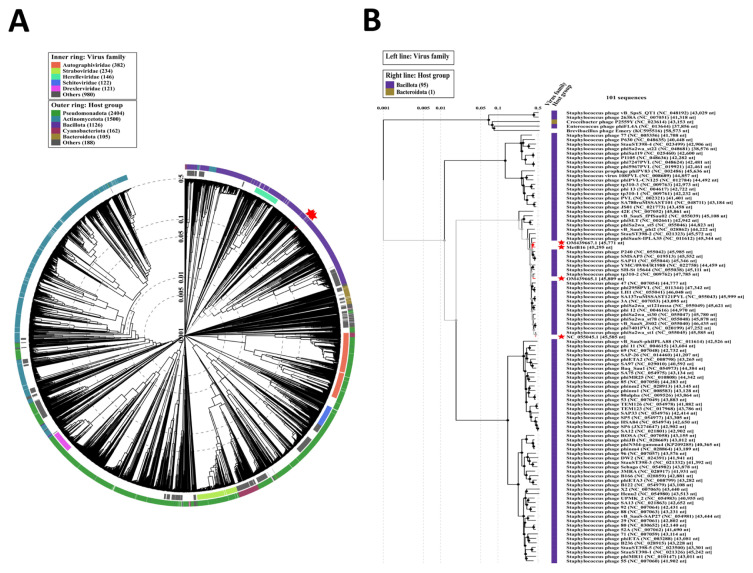
Proteomic tree of phage MetB16 (A) Circular proteomic tree displaying genome-wide similarities among phage MetB16 (highlighted with a red star), top BLASTn matches, and closely related reference phage genomes. (B) Rectangular proteomic tree illustrating phage MetB16 and the top 100 phages with the highest ViPTree SG scores.

We utilized VipTree for proteome-based phylogenetic analysis, comparing the MetB16 genome with approximately 5636 phages. Notably, neither MetB16 nor its closest relatives (NC_055045.1, OM439665.1, and OM439667.1) clustered with phages whose bacterial hosts belong to the class Bacillota (Figure 5A). Subsequently, we selected MetB16 and the top 100 phages with the highest VipTree SG scores to construct a rectangular proteomic tree (Figure 5B, Table S6).

**Figure 6:**
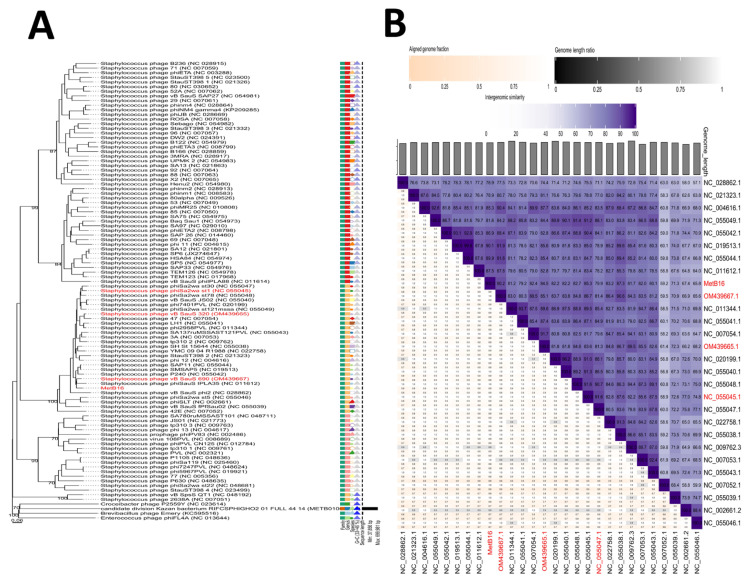
Phylogeny of phage MetB16. (A) The VICTOR genome-based phylogenetic tree was constructed by comparing phage MetB16, other *Caudoviricetes* phages, and closely related phages using Viptree. The phages were organized into 2 families and 7 genera, further grouped into 95 species. The GC content of the phage genomes is depicted in varying shades of purple, while the genome size is represented by horizontal black lines on the right side. (B) The VIRIDIC heatmap illustrates the intergenomic similarity between phage MetB16 and closely related phages, as classified by VICTOR. Three alignment capacity factors were considered to calculate the relatedness between the phages: intergenomic similarities (shown in hues of purple to lilac), the percentage of aligned genome sequence in a pair (indicated by shades of pink), and their length ratio (displayed in shades of black).

Phylogenetic analysis using VICTOR with the GBDP method identified 95 species clusters, seven genus clusters, and two families, with an average support of 14%. VICTOR grouped MetB16 with other closely related phages in the family unclassified *Siphoviruses*, at the same genus level (Figure 6A). Within this grouping, MetB16 was identified as a distinct and unique species. Intergenomic distances and similarities among the phages identified by VICTOR as belonging to the same genus as MetB16 were computed using VIRIDIC. This analysis clustered MetB16 and 27 other phages into 26 species clusters, with most phages assigned to distinct species, except for four (NC_019513.1, NC_055044.1, NC_020199.1, NC_055040.1). Of these, NC_019513.1 and NC_055044.1 were also classified into the same genus by VICTOR. VIRIDIC categorized the phages into three distinct genera: NC_007052.1 constituted one genus, NC_055039.1, NC_002661.2, and NC_055046.1 comprised another distinct group, and the remaining phages, including MetB16, formed a separate genus. The intergenomic similarities among phages MetB16, NC_019513.1, NC_055044.1, and OM439667.1 were 90.1%, 89.9%, and 90.2%, respectively (Figure 6B). These similarities reflect the highest similarity within the same genus, aligning with VIRIDIC’s predefined thresholds of 95% for species and 70% for genus classification.

These findings strongly indicate that phage MetB16 belongs to the same genus as 27 *Triaviru*s phages, though it is not within the same species. Therefore, we conclude that phage MetB16 should be classified as representing a new species within the class *Caudoviricetes*.

### 3.5. Modeling of therapeutic proteins

**Figure 7:**
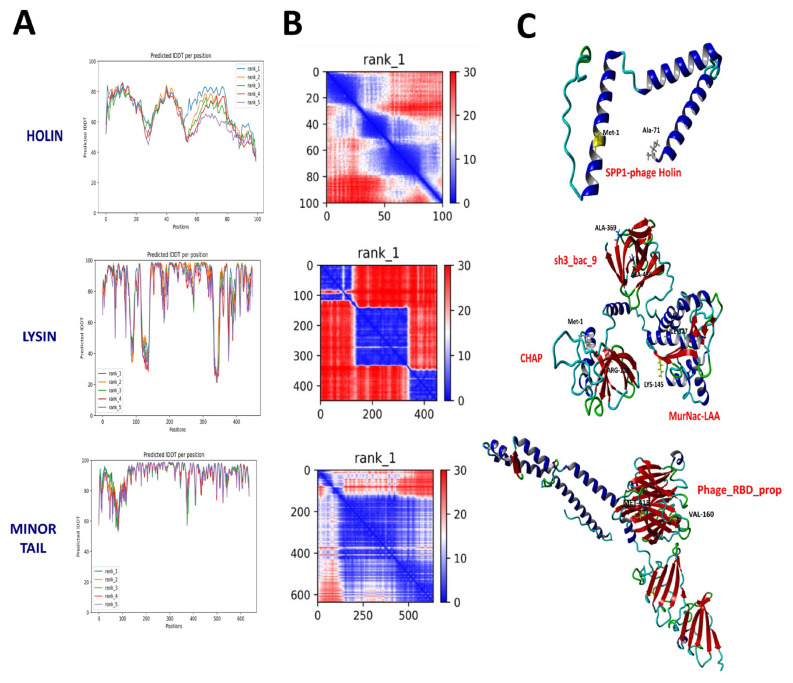
Modeling of MetB16 Holin, Lysin and Minor Tail Protein. (A) Predicted IDDT per position. Residues scoring ≥90 on pIDDT indicate an exceptionally high confidence level, while those scoring between 90 and 70 are deemed to have high confidence. Residues scoring between 70 and 50 on the pLDDT metric exhibit low confidence, and those below 50 indicate extremely low confidence. (B) The Prediction Aligned Error (PAE) score for the top-ranked model illustrates the calculated error in predicted distances between pairs of residues. The positional indices of individual amino acids are represented on both axes. The uncertainty in the predicted distance between any two amino acids is color-coded from blue (0 Å) to red (30 Å), as depicted in the accompanying color bar. (C) New cartoon representation model illustrating the structure of proteins incorporates the pLDDT scores per position. These scores are integrated as the b-factor in the PDB file provided by AF2, represented in rainbow colors ranging from red (indicating high confidence) to blue (suggesting low confidence). Based on Interpro domain predictions, the pertinent residue is highlighted in black, while the domains are marked in red.

**Figure 8:**
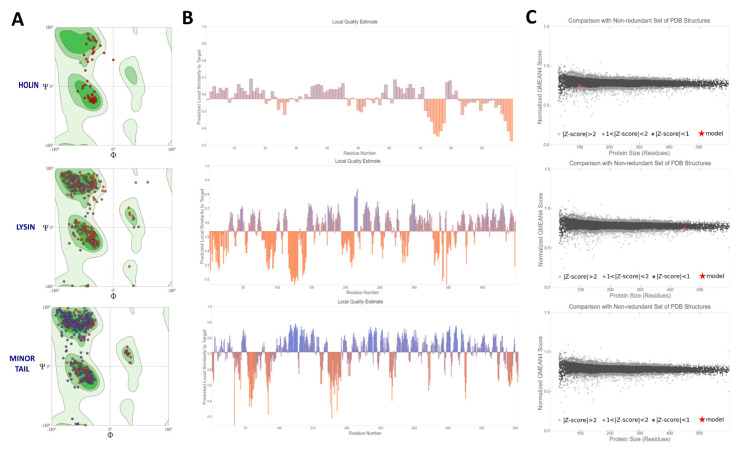
Structure Assessment of MetB16 Holin, Lysin and Minor Tail Protein. (A) The Ramachandran plot of the predicted structure from top ranked models illustrates the distribution of phi (φ) and psi (ψ) dihedral angles for each residue in the protein. This plot provides insights into the conformational quality and stereochemical accuracy of the protein models. Light green indicates that 99.7% of the data falls within the first contour line, medium green represents 95.0% within the second contour line, and dark green signifies 80.0% within the third contour line. (B) Each residue in the model is assigned a local quality score (x-axis), indicating its expected similarity to the native structure (y-axis). Residues with scores below 0.6 are typically indicative of low quality. (C) Plot is represented by the normalized QMEAN score (y-axis), contrasted with scores attained by high-resolution crystal structures. Elevated values suggest that the model shares a comparable quality to experimental structures of similar dimensions.

While PHOBIUS detected a critical signal sequence for recombinant production (Table S7), this was not confirmed by SignalP 6.0 or TOPCON (data not shown). The MetB16 lysin protein was characterized by three domains: residues 1–113 corresponded to the CHAP domain (IPR007921), residues 145–327 to the MurNAc-LAA domain (IPR002508), and residues 369–436 to the sh3_bac_9 domain (IPR003646) (Figure S3A, Table S8, Figure 7). Furthermore, the region spanning residues 160–418 of the Minor Tail Protein sequence contains the Phage 5-bladed beta-propeller receptor binding platform (PF21311), which is identical to the domain found in gp45, the Receptor Binding Protein (RBP) of the well-studied phi 11 phage (Figure S3B, Table S9, Figure 7). Functional domain analysis of the MetB16 holin protein, using InterPro, identified similarities to holins from tailed bacteriophages (IPR006479) (Figure S3C, Figure 7). AlphaFold2 (AF2) was used to model the 3D structures of the MetB16 holin, lysin, and minor tail proteins. For lysin, AF2 utilized various PDB templates (1jwq, 2mk5, etc.), while the minor tail protein was modeled with PDBs 5efv, 6iab, and 6v8i. The predicted structures, rated on a scale from 0 to 100 for each residue, are shown with atomic coordinates and confidence scores (pIDDT) (Figure 7A). High-confidence regions of the lysin and minor tail protein models were close to a score of 100, although Prediction Aligned Error (PAE) graphs indicated low confidence in certain areas (Figure 7B). The average pLDDT score for the holin prediction was notably low, reflecting lower reliability (Figure 7C). The structural evaluation of the AF2 models was performed using the Swiss Model Structure Evaluation tool and MolProbity. Swiss Model assessed the small tail protein structure prediction as successful, while the holin and lysin models required improvements (Figure 8 A, B, C). MolProbity analysis indicated that the holin and lysin models did not meet Ramachandran’s preferred cutoff (Table S10). To enhance model accuracy, homology modeling was conducted with Swiss Model using Q2FYD7.1.A as a template for the holin, achieving a GMQE of 0.66. Quality assessment showed that 92.9% of residues were in favored regions and 100.0% in allowed regions, representing a significant improvement (Table S11). The AF2 model more accurately predicted the membrane binding area compared to the Swiss-Model holin model (Figure S4). Additionally, the AF2 model’s Z value for holin surpassed that of the Swiss-Model prediction (Figure S4, Table S12). In homology modeling for lysin, Swiss-Model utilized A0A2A1K7F1.1.A as a template with a sequence identity of 72.20%. According to the MolProbity report, model derived from the Swiss-Model lysin estimate exhibited superior structure in terms of various parameters, except for the bad angles value (Table S13). The AF2 minor tail protein was evaluated as successful across all parameters except for the estimated bad angle value (Table S14).

## 4. Discussion

Bacterial strains resistant to antibiotics, arising from the evolution of bacteria to evade the effects of antibiotics, can occur through horizontal gene transfer processes such as conjugation, transformation, and transduction (Balcázar, 2020; Pfeifer et al., 2022). Phage-mediated transduction is a known phenomenon in the spread of antibiotic resistance genes among staphylococci (Frosini et al., 2020; Borodovich et al., 2022). *S. aureus*, with its production of numerous toxins and virulence factors, exhibits opportunistic pathogenic characteristics, invading its host and causing diseases that have been treated with antibiotics for many years. However, strains of *S. aureus*, such as MRSA and Vancomycin-resistant *Staphylococcus aureus* (VRSA), have developed resistance to antibiotics over time due to intense and indiscriminate antibiotic use. These resistant strains are globally significant contributors to nosocomial infections, posing one of the most significant threats to human health (Livermore and Woodford, 2006; Boucher, 2020; Hekimoğlu et al., 2022). Both the potential use of phages as therapeutic agents in the treatment of bacterial infections through alternative treatment approaches and the understanding that phages may also play a role in the causation of this resistance make isolations, characterizations, and genomic analyses highly essential. The genomic integration of temperate phages not only draws attention in terms of the potential traits they can confer to their hosts but also opens the door to understanding the coevolution of bacteria and phages. Additionally, advancements in synthetic biology today have paved the way for the use of temperate phages in phage therapy, making them intriguing due to their unique features (Monteiro et al., 2019). In this study, we isolated the temperate phage Staphylococcus phage vB_Sau_MetB16 from MRSA strain and performed characterization and genome analysis of this phage.

Following a dense plaque observed in the spot test conducted after 60 s of UV light exposure of the MRSA culture, the titer of MetB16, determined by the double agar overlay technique, was found to exhibit a morphological structure specific to the *Siphoviridae* family, which is updated by ICTV, in STEM analysis, confirming the genomic analysis results (Hyman and Abedon, 2009; Turner et al., 2023). While its mechanism is largely unclear and influenced by various factors, it is a widely accepted notion that lytic phages exhibit a low optimum MOI value, whereas lysogenic phages have a high optimum MOI value (Zhang et al., 2022). To explain this mechanism, Golding et al., propose the following pathway; temperate phages, after introducing their genomic materials to the hosts, assess the density of the host in the environment. If the host is scarce in the environment, continuing the lytic cycle would be inefficient, and therefore, a high MOI value is associated with lysogeny for this reason (Golding et al., 2021; Yao et al., 2021). The optimal MOI found for metB16 is equal to 1 and can be considered a relatively high value (Konopacki et al., 2020; Mantlo et al., 2020). By defining the host range of a phage, it is important to use the term “broad host range” accurately. Ross et al. have indicated that this term can generally be used for phages capable of infecting multiple bacterial species as well as different strains of the same species. Therefore, it can be stated that MetB16 has a broad host range (Ross et al., 2016). Because, in a manner consistent with the results obtained from genomic analysis, it has been determined via host range analysis that MetB16 can infect not only various *S. aureus* strains but also a different bacterial species, *Bacillus subtilis and Staphylococcus chromogenes* (respectively EOP values; 6.92×10^−7^ and 1.31×10^−7^). Phage growth characteristics, including phage adsorption rate, latent period, burst size, stationary phase, and lysis time, were examined by adsorption curve and one-step growth analysis (Kaur et al., 2012). The lysis period of MetB16, which had a latent period of 20 min, lasted approximately 45 min and its burst size was determined as 127 PFU/infected cells. Programming of host cell lysis is critically controlled by the holin protein expressed by phages. High burst size, which plays a very important role in phage propagation, is an indicator of an active lysis effect. Induced temperate phages can produce much higher burst sizes than lytic phages (Liu et al., 2022). The life cycle of MetB16 was similar to that of other *Staphylococcus* phages Stau2, IME-SA1, and SaGU1 (burst sizes; 100, 80, and 117 PFU/cell, respectively) (Shimamori et al., 2021). In the study conducted by Abdurahman et al. with four different temperate *S. aureus* phages, they found that the phages belonged to the *Siphoviridae* family, reached burst size in 50 min following a latent period of approximately 20 min, and stated that these four phages could be used as potential agents for use in phage therapy. Temperature is critical in determining the viability and lytic activity of phages, which consist of a protein coat surrounding their genetic material. Denature of the protein as the temperature increases will affect the stability of the phage, its adsorption process to its host, and self-replication (Ackermann et al., 2004; Jończyk et al., 2011; Wen et al., 2023). At the same time, the pH of the environment or to which the phage is exposed also plays a critical role in the life of the phage. Temperature and pH stability of MetB16 was evaluated by short-term incubation. It has been observed that the MetB16 phage begins to lose its lytic activity as temperatures rise above 50 °C, confirming the thermal stability and pH stability of phages belonging to the *Siphoviridae* family, and maintains its viability in the pH range of 4–12 (Jończyk et al., 2011; Jamal et al., 2015; Litt and Jaroni, 2017; Selcuk et al., 2024a)

Several tools were employed to analyze the whole genome sequence of phage MetB16, and all analyses consistently indicated the absence of known genes encoding virulence factors or antimicrobial resistance. PhageAI platform predicts a high probability of lysogenic potential for phage MetB16. While the therapeutic potential of temperate phages remains less explored compared to phages with a lytic life cycle, the significance of temperate phages’ antimicrobial peptides cannot be overlooked despite limitations in current research on phage therapy. Furthermore, the analysis revealed the presence of genes encoding potential antimicrobial peptides, among which holins exhibited predicted topologies consistent with class II holins (Labrie et al., 2004; Abeysekera et al., 2022). Given that MetB16 was isolated from a clinical sample, it makes sense to opt for using phage antimicrobial peptides in patient-specific treatments, as these peptides offer an advantage by preventing bacterial resistance (Mirski et al., 2019; Nandi et al., 2022).

In the genome of phage MetB16, a cluster of adjacent genes encoding endolysin-holin was identified, along with a Clp protease-like protein outside this cluster. This gene arrangement aligns with the conical lysis mechanism typical of tailed phages at the end of their lytic cycle (Oliveira et al., 2019). Holins create micron-scale pores in the cytoplasmic membrane of Gram-positive bacteria, allowing endolysins to access and degrade the peptidoglycan layer. Literature supports the use of phage-encoded EPS depolymerases to disrupt staphylococcal biofilms (Gutiérrez et al., 2015a; Gutiérrez et al., 2015b, a; Save et al., 2022). The MetB16 minor tail protein (ORF 21) shows significant similarity to hypothetical proteins in various *Staphylococcus* strains, such as WP_000429551.1, annotated as a minor structural protein. Comparative analysis revealed identical minor tail protein sequences in several *Staphylococcus* phages, including phi 12, tp310-2, StauST398-2, and phiSa2wa_st121mssa. Notably, the MetB16 minor tail protein also shares homology with the *E. coli* protein MQL15017.1 (e-value 0, 100% identity, 100% query cover). While this homology does not fully explain a broad host range (Kang et al., 2017; Song et al., 2019; Loh et al., 2020), it suggests a potential link between MetB16 and various Staphylococcus species, supported by host range studies.

Phage phylogenetic analysis is complicated by genomic mosaicism due to frequent mutations, horizontal gene transfer, and the absence of universally conserved genes. (Zaki et al., 2023). Phage MetB16 was analyzed using multiple methodologies, as recommended (Grose and Casjens, 2014; Ramy et al., 2019; Zaki et al., 2023). Despite Viptree’s limitations in determining MetB16’s family, data from NCBI blastn, VIRIDIC, and VICTOR indicate that MetB16 is related to *Caudoviricetes* class phages within the *Triavirus* genus, consistent with electron microscopy findings. However, none of these tools suggest MetB16 belongs to the same species as known Triaviruses. Therefore, phage MetB16 is proposed as a new species within the *Triavirus* genus. Additionally, temperate phages facilitating horizontal gene transfer (HGT) can mediate the dissemination of mobile genetic elements, including toxin genes, virulence island gene clusters, and antibiotic resistance determinants, among bacterial populations. Nevertheless, the absence of virulence factors or antimicrobial resistance genes in the genome of the MetB16 phage underscores its potential as a safe and promising candidate for biotechnological applications. This finding mitigates concerns associated with the horizontal transfer of deleterious genetic material, thereby positioning MetB16 as a viable therapeutic and industrial tool.

Concerns about the therapeutic potential of temperate phages arise due to their evolutionary advantageous relationship with their host (Loc-Carrillo and Abedon, 2011; Lin et al., 2022). Nevertheless, like lytic phages, temperate phages possess therapeutically valuable proteins. This study focused on enhancing the 3D structure predictions of AF2 by examining the structural features of holin, endolysin, and the minor tail protein crucial for MetB16’s lifecycle (Glazer et al., 2009; Adiyaman et al., 2023). Future studies employing high-resolution techniques such as nuclear magnetic resonance (NMR), X-ray crystallography, and cryo-electron microscopy (cryo-EM) to analyze these proteins’ structures will address a significant gap in the literature. Our preliminary findings suggest that improving the physicochemical properties of these structures can advance the use of MetB16’s temperate phage proteins in therapeutic and targeting applications.

## 5. Conclusion

This study investigates the isolation, structural and growth characteristics, as well as the genomic and proteomic features of a temperate phage targeting MRSA, a significant issue due to its antibiotic resistance. By examining the biology and genetics of these phages, we aim to evaluate their potential as alternative therapeutic options. Phage MetB16 has been classified at the genus level with a broad range of activity and lacks known antibiotic resistance genes. It was endeavored to elucidate the structures of therapeutically valuable holin, endolysin, and tail proteins. We have elucidated the structures of key therapeutic proteins holin, endolysin, and tail proteins. These findings suggest that MetB16 can be employed in synergistic treatment approaches and targeted infection site therapies. This research provides comprehensive data that enhances our understanding of phage-host interactions and offers valuable insights for evaluating temperate phages in phage therapy.

## Supplementary information

Supplementary tables are deposited to the APERTA database, accessible at https://aperta.ulakbim.gov.tr/record/286002

Supplementary Figure S1 Raw sequencing reads map. Red and green lines represent the reads. It is represented matched phage sequence is dark and unmatched is light.

Supplementary Figure S2 Predicted topology of ORF 26 (WWP17606.1) (putative class II holins). DeepTMHMM was used to predict the topology of ORF 26. The top part of each chart represents the topology of predicted domains in correspondence to the amino acid sequence: transmembrane (in red), intracellular (in pink), and extracellular (in blue). The probability of predicted topology is presented in the bottom part. The topology of holin in ORF 26 was predicted by 100%.

Supplementary Figure S3 InterPro domain analysis of Therapeutic Proteins. (A) MetB16 lysine, (B) minor tail protein, (C) holing.

Supplementary Figure S4 Structure Assessment of Swiss-Model Prediction MetB16 holin’s and AF2 holin membrane localization prediction. (A) Per residue/Local quality score. (B) The normalized QMEAN score. (C) The Ramachandran plot of the predicted structure from top ranked model. (D) New cartoon representation holin model and InterPro domain prediction residues. (E) The AF2’s holin structure prediction was assessed as a transmembrane protein using Structure Assessment.

## Data Availability

All relevant data generated or analyzed during this study are included in this article. The complete genome sequences of phage MetB16 and *S. aureus* MetB16 were deposited into GenBank, and the accession number are PP357935 and CP162608, respectively.
