# Supplementary figures and images for: Novel MRSA-targeting phage MetB16: Genomic features, structural insights, and therapeutic applications
Source: Turk J Biol. 2025 Feb 14;49(3):292–308. doi: 10.55730/1300-0152.2746 (PMC12266354; doi:10.55730/1300-0152.2746)

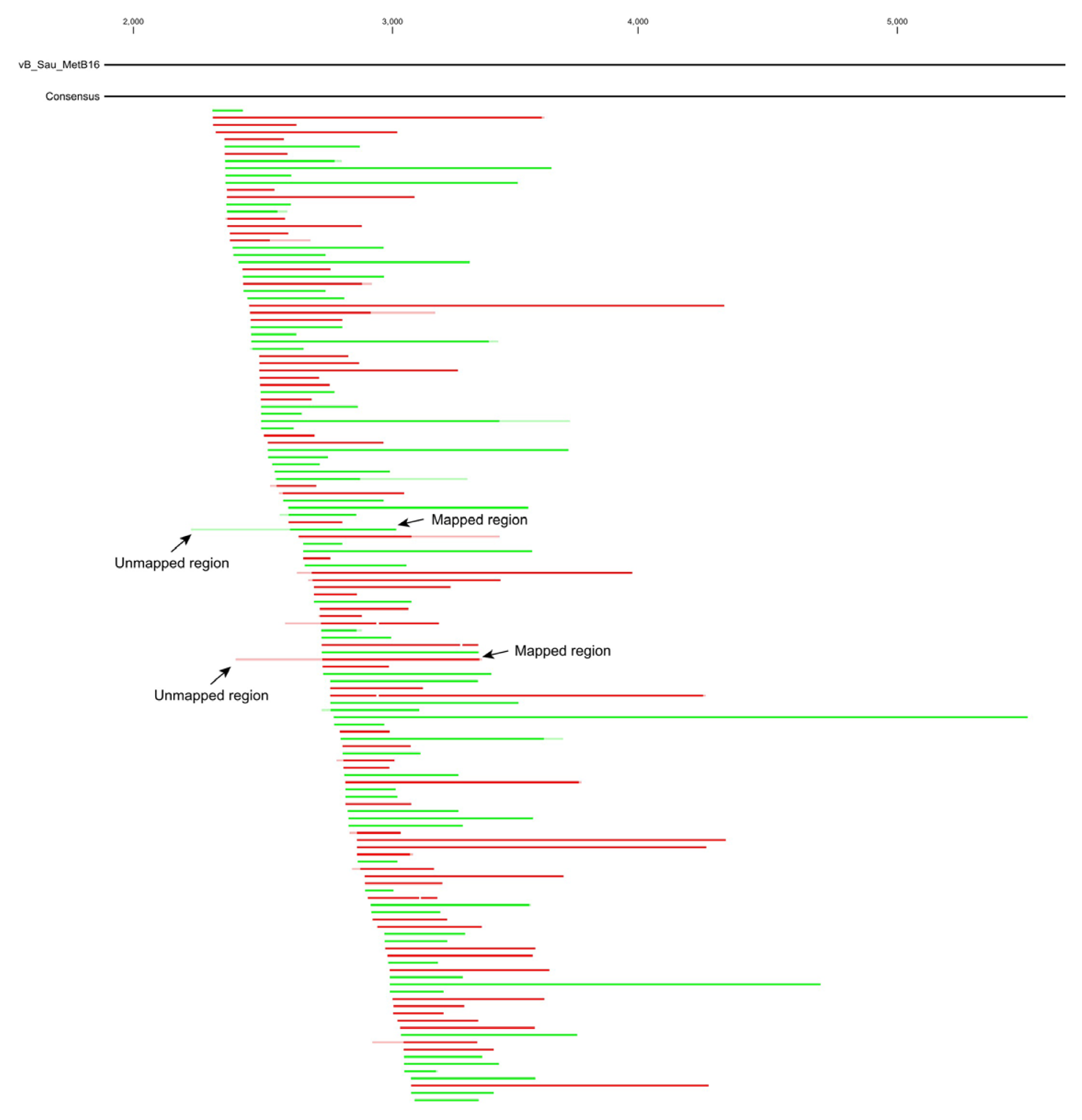

Supplement: Supplementary Figure S1 — Raw sequencing reads map. Red and green lines represent the reads. It is represented matched phage sequence is dark and unmatched is light. [file tjb-49-03-292s1.tif]

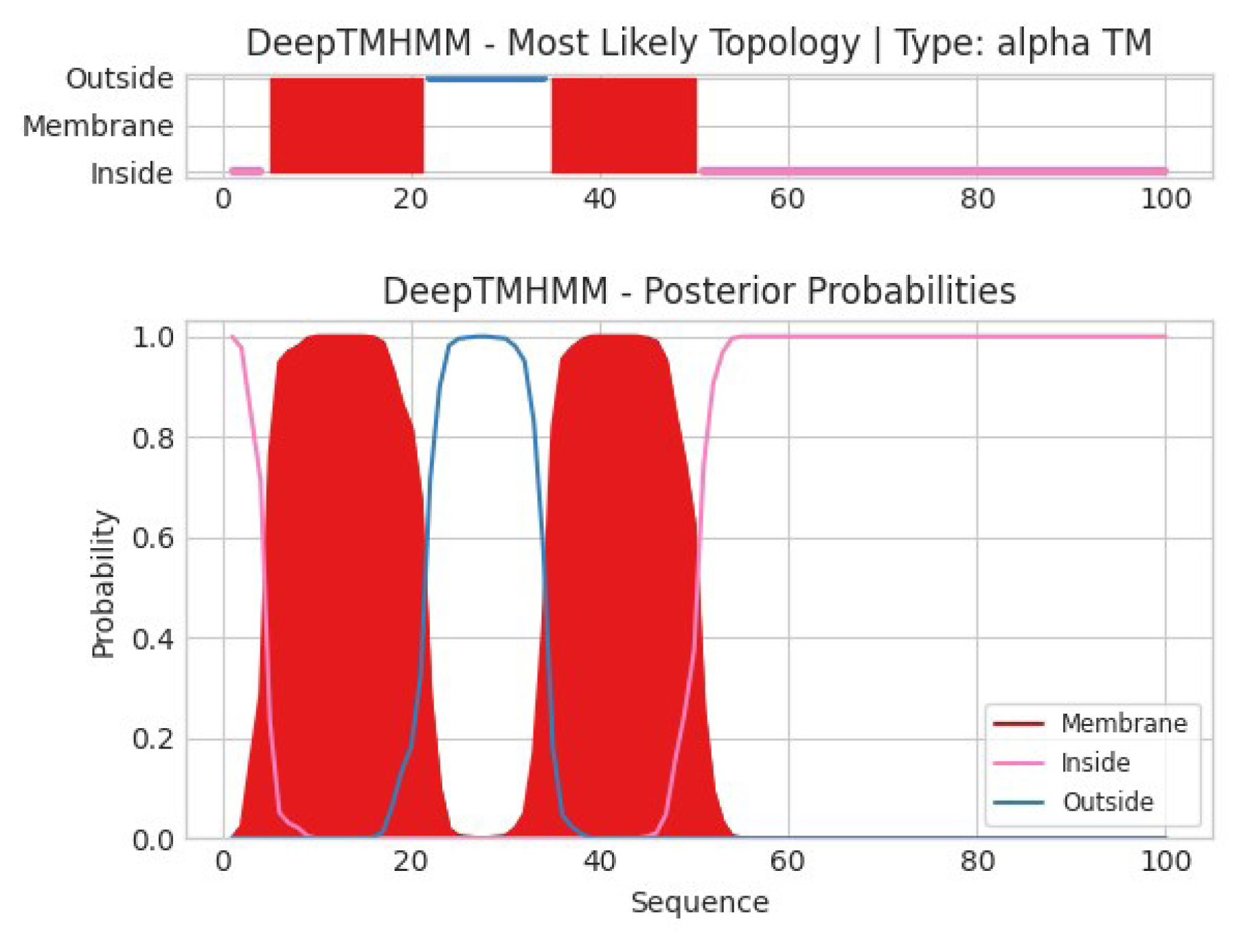

Supplement: Supplementary Figure S2 — Predicted topology of ORF 26 (WWP17606.1) (putative class II holins). DeepTMHMM was used to predict the topology of ORF 26. The top part of each chart represents the topology of predicted domains in correspondence to the amino acid sequence: transmembrane (in red), intracellular (in pink), and extracellular (in blue). The probability of predicted topology is presented in the bottom part. The topology of holin in ORF 26 was predicted by 100%. [file tjb-49-03-292s2.tif]

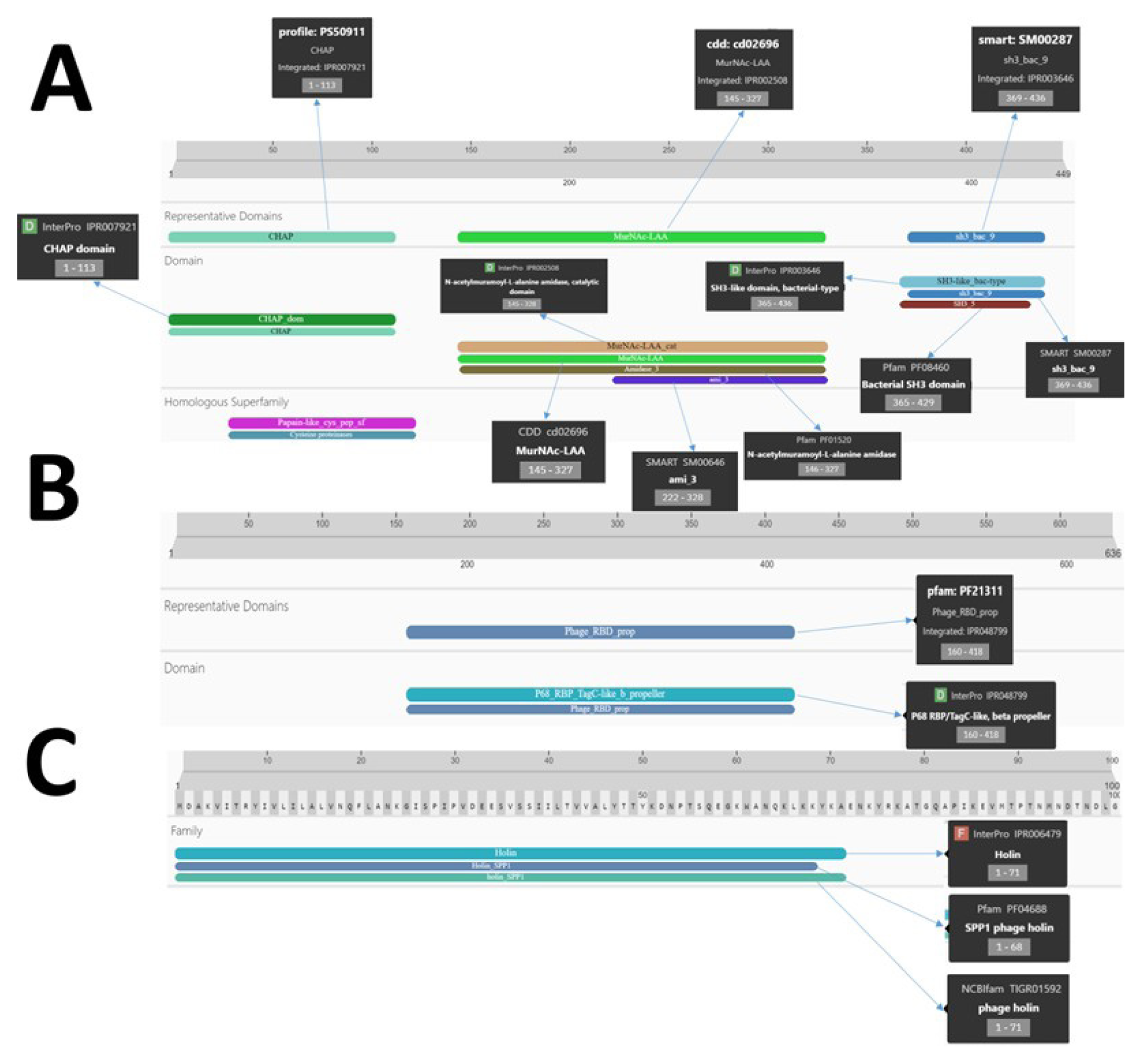

Supplement: Supplementary Figure S3 — InterPro domain analysis of Therapeutic Proteins. (A) MetB16 lysine, (B) minor tail protein, (C) holing. [file tjb-49-03-292s3.tif]

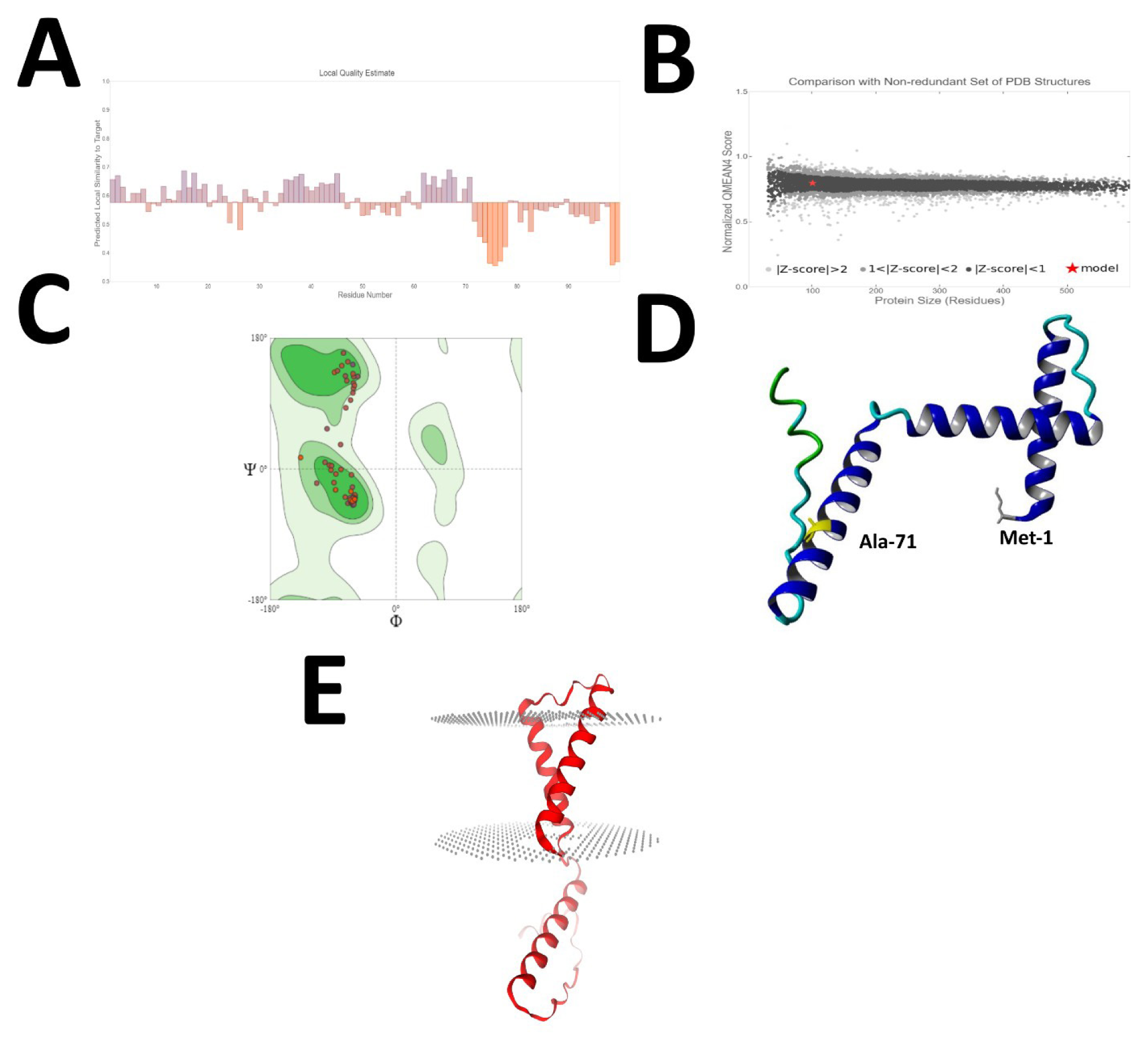

Supplement: Supplementary Figure S4 — Structure Assessment of Swiss-Model Prediction MetB16 holin’s and AF2 holin membrane localization prediction. (A) Per residue/Local quality score. (B) The normalized QMEAN score. (C) The Ramachandran plot of the predicted structure from top ranked model. (D) New cartoon representation holin model and InterPro domain prediction residues. (E) The AF2’s holin structure prediction was assessed as a transmembrane protein using Structure Assessment. [file tjb-49-03-292s4.tif]
